# Abrogating mitochondrial ROS in neurons or astrocytes reveals cell-specific impact on mouse behaviour

**DOI:** 10.1016/j.redox.2021.101917

**Published:** 2021-03-03

**Authors:** Carlos Vicente-Gutierrez, Nicolo Bonora, Daniel Jimenez-Blasco, Irene Lopez-Fabuel, Georgina Bates, Michael P. Murphy, Angeles Almeida, Juan P. Bolaños

**Affiliations:** a Institute of Functional Biology and Genomics, University of Salamanca, CSIC, 37007, Salamanca, Spain; b Centro de Investigación Biomédica en Red Sobre Fragilidad y Envejecimiento Saludable (CIBERFES), Instituto de Salud Carlos III, Madrid, Spain; c Institute of Biomedical Research of Salamanca, University Hospital of Salamanca, University of Salamanca, CSIC, 37007, Salamanca, Spain; d MRC Mitochondrial Biology Unit & Department of Medicine, University of Cambridge, Cambridge, CB2 0XY, United Kingdom

**Keywords:** Mitochondria, ROS, Neuron, Astrocyte, Signallling, *In vivo*

## Abstract

Cells naturally produce mitochondrial reactive oxygen species (mROS), but the *in vivo* pathophysiological significance has long remained controversial. Within the brain, astrocyte-derived mROS physiologically regulate behaviour and are produced at one order of magnitude faster than in neurons. However, whether neuronal mROS abundance differentially impacts on behaviour is unknown. To address this, we engineered genetically modified mice to down modulate mROS levels in neurons *in vivo*. Whilst no alterations in motor coordination were observed by down modulating mROS in neurons under healthy conditions, it prevented the motor discoordination caused by the pro-oxidant neurotoxin, 3-nitropropionic acid (3-NP). In contrast, abrogation of mROS in astrocytes showed no beneficial effect against the 3-NP insult. These data indicate that the impact of modifying mROS production on mouse behaviour critically depends on the specific cell-type where they are generated.

## 1 Introduction

The high energy requirement of neurotransmission is sustained by integrating different metabolic programs of the brain cells [1]. For instance, astrocytes, which have direct access to the bloodstream glucose, mostly rely on glycolysis to obtain energy, whereas neurons depend mainly on oxidative phosphorylation [2,3]. This metabolic organization allows astrocytes to supply glycolytically-derived lactate to neurons as a readily oxidizable substrate [4]. In good agreement with these observations, mitochondria are better coupled [5] and produce about one order of magnitude less mitochondrial reactive oxygen species (mROS) [6] in neurons than in astrocytes. This vast difference in mROS production is accounted for by the assembly configuration of the mitochondrial respiratory chain supercomplexes [7]. Thus, this configuration is tight in neurons to ensure a high energy efficiency, which results in a weaker electron flow to oxygen to form mROS by complex I than that observed in astrocytes [6]. Actually, the production of mROS by astrocytes is physiologically high in order to sustain brain complex functions such as behaviour [8], in good agreement with the notion that mROS are signalling molecules participating in several cellular processes [9]. Conversely, high levels of mROS in neurons are often associated with pathology [10]. These observations suggest that understanding the biological or pathological functions of ROS should be gathered by dissecting out the specific cell type where they are produced and exert their actions. Here, we aimed to investigate the cellular and behavioural impact of neuronal mROS abundance both under physiological and pathological circumstances.

## 2 Results and discussion

**Fig. 1:**
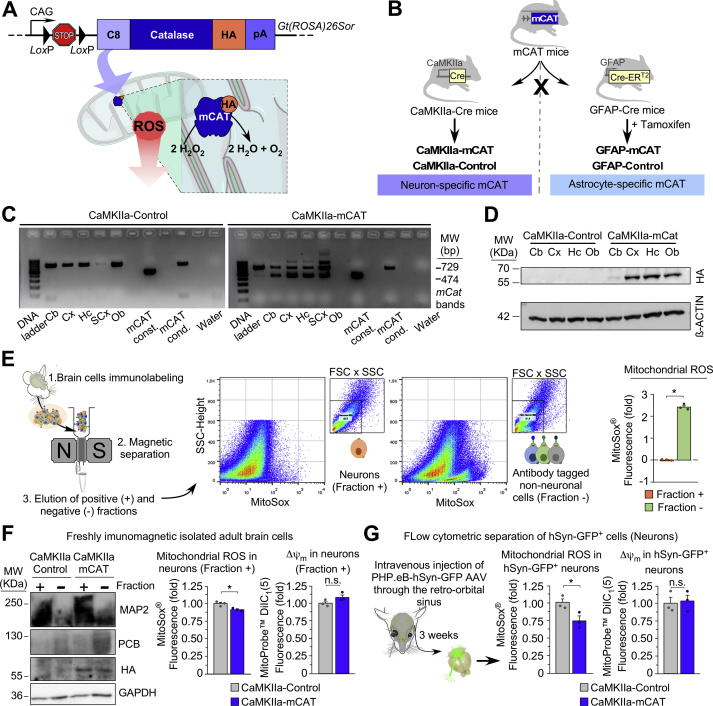
Design and generation of transgenic mice to downmodulate mitochondrial ROS cell-specifically *in vivo*. (*A*) Schematic of the genetic strategy used to generate the conditional mCAT^*LoxP*^/+ mouse. (*B*) Strategy to generate mice expressing mCAT in neurons or astrocytes. (*C*) Detection of the Cre-mediated recombination of the mCAT allele (474 bp) by PCR in different brain regions of CaMKIIa-mCAT mice (Cb, cerebellum; Cx, cortex; Hc, hippocampus; SCx, sub-cortical area; Ob, olfactory bulb). (*D*) Occurrence of HA-tagged mitochondrial catalase as judged by western blotting. (*E*) Schematic workflow on the left represents immunomagnetic separation of neuronal-enriched and non-neuronal enriched (mainly glial) cell fractions from wild type animals, followed by flow cytometric analysis of both cell fractions with MitoSox (n = 3 mice). (*F*) Western blot and flow cytometric analyses of immunomagnetically-purified neuronal positive fractions showing HA-tagged mitochondrial catalase in mouse-derived samples enriched in neuronal (MAP2-positive) fractions (left panel, n = 2 mice), and mROS in neuronal-enriched fractions (right panel; n = 3 mice). (*G*) Flow cytometric analysis of cells dissociated from mouse brains 3 weeks after intravenous administration of hSyn-promoter-driven GFP AAVs showing mROS in the GFP positive population (neurons) (n = 3 mice). *p < 0.05.

To downregulate endogenous mROS abundance *in vivo* in a cell-specific manner, we used a mitochondrial-tagged catalase (mCAT) inducible mouse model previously generated in our laboratory [8]. This mouse harbours, in the *Rosa26* locus, the full-length cDNA of catalase fused to the cytochrome *c* oxidase subunit VIII–mitochondrial leading sequence (C8) and to the human influenza haemagglutinin (HA) for tagging purposes (Fig. 1*A*). A floxed (*LoxP*-flanked) transcriptional STOP cassette was incorporated between the mCAT cDNA and the CAG promoter (mCAT^*LoxP*^/+) (Fig. 1*A*). This system allows mCAT expression *in vivo* cell-specifically, otherwise under the control of the same strong CAG promoter. The efficacy of the mCAT system to downmodulate mROS has been previously validated [8]. In order to downmodulate mROS in neurons, mCAT^*LoxP*^/+ mice were mated with mice harbouring the Cre recombinase governed by the neuronal-specific promoter of mouse calcium-calmodulin kinase IIa (CaMKIIa-Cre/+) (Fig. 1*B*, left panel). The resulting progeny were CaMKIIa-Cre/+; mCAT/+ (CaMKIIa-mCAT) and +/+; mCAT^*LoxP*^/+ littermates (CaMKIIa-Control) (Fig. 1*B*, left panel).

The effective recombination of the mCAT inducible allele was confirmed by PCR (Fig. 1*C*) and by western blotting against the HA epitope (Fig. 1*D*) in different brain regions of the CaMKIIa-mCAT mice. To confirm that mCAT expression in CaMKIIa-mCAT mice downmodulates mROS specifically in neurons, neurons were isolated from adult mice using an immunomagnetic labelling system that separates unlabelled (neurons) from immunolabelled non-neuronal (mainly glial) cell fractions (Fig. 1*E*). Flow cytometric analysis of MitoSox fluorescence in both cell fractions confirmed less mROS abundance in the neuron-enriched fraction (Fig. 1*E*) (see also ref. [6]). Notably, the MitoSox fluorescence signal was lower in the neuron-specific marker microtubule-associated protein-2 (MAP2)-positive cells isolated from the CaMKIIa-mCAT when compared with the CaMKIIa-Control mice (Fig. 1*F*). To further confirm this, we used a different approach in which mice were intravenously injected, through the retro-orbital sinus, with adeno-associated viruses (AAVs) expressing green fluorescent protein (GFP) under the control of the human neuron-specific synapsin (hSyn) promoter (Fig. 1*G*). Flow cytometric analysis of the brain cell suspensions obtained from these mice confirmed a ~25% reduction in the MitoSox fluorescence signal in the GFP-positive cell population (neurons) of the AAV-injected CaMKIIa-mCAT mice (Fig. 1*G*).

**Fig. 2:**
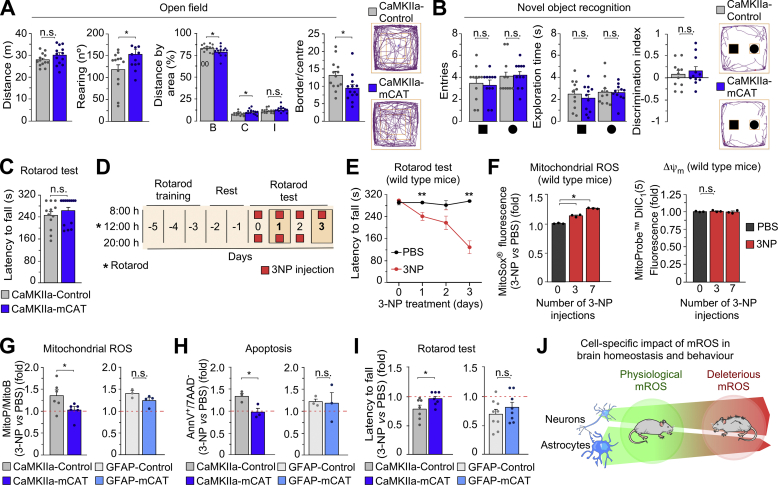
Behavioural impairment by 3-NP treatment is abolished by abrogating neuronal, not astrocytic mROS. (*A*) Registered movement tracks in the open field test showing the distance travelled, number of rearing and the ratio of the time spent in the periphery *versus* the time spent in the centre areas (B, border; C, centre; I, intermediate; n = 13 male mice per genotype; *p < 0.05). (*B*) Novel object recognition test showing the number of entries, the exploring time spent with the novel object (sphere) and the discrimination index (n = 11 male mice per genotype; n. s., not significant). (*C*) Motor coordination analysis according to the rotarod test (n = 12–13 mice per genotype). (*D*) Schematic representation of the rotarod test protocol to assess motor discoordination induced by 3-NP (50 mg/kg of body weight; i. p.) or vehicle (phosphate-buffered saline, PBS). (*E*) Evaluation of motor coordination by rotarod test upon 3-NP treatment (n = 6 male mice per condition; *p < 0.05 *vs* corresponding PBS). (*F*) *Ex vivo* measurement of mitochondrial ROS by flow cytometry in adult brain cells with MitoSOX upon 3-NP treatment (n = 2–3 male mice per condition and genotype; *p < 0.05 and n. s. *vs* the corresponding PBS condition indicated by the dotted red line). (*G*) *Ex vivo* measurement of mitochondrial H_2_O_2_ with the ratiometric mass spectrometry MitoB probe, which accumulate within the mitochondria to react with H_2_O_2_ forming MitoP. Mice were previously injected stereotaxically with 3-NP (right hemisphere), PBS (left hemisphere) and MitoB (lateral ventricle) 6 h before the sample extraction. (*H*) *Ex vivo* measurement of apoptotic cell death by flow cytometry in adult brain cells upon 3-NP treatment (the dotted red line represents PBS-treated mice) (n = 2–3 male mice per condition and genotype; *p < 0.05). (*I*) Evaluation of motor coordination by rotarod test upon 3-NP treatment (the dotted red line represents PBS-treated mice) (n = 7–9 male mice per genotype; *p < 0.05). (*J*) Schematic summary of the main message of this study. (For interpretation of the references to colour in this figure legend, the reader is referred to the Web version of this article.)

Next, we evaluated the spontaneous behaviour of male CaMKIIa-mCAT mice. The open field test showed no alteration in the distance travelled but increased the ratio of the time spent in the centre *versus* the border and the rearing number (Fig. 2*A*). The novel object recognition (Fig. 2*B*) and the rotarod (Fig. 2*C*) tests showed no changes, suggesting that, under normal conditions, neuronal mROS is not involved in the control of exploration, short-term memory or motor coordination in the mouse. However, it is known that increased mROS levels in the brain mediate neurological diseases [11], although the repercussion that mROS generated by each cell type on neural impairment is unknown. We therefore sought to compare the contribution of neuronal and astrocytic mROS to the organismal response to a pro-oxidant neurotoxic challenge. To do so, we used the CaMKIIa-mCAT mouse model herein characterized to prevent the increases in mROS in neurons. To prevent astrocytic mROS enhancements, we mated the mCAT^*LoxP*^/+ mice with mice harbouring the Cre recombinase governed by the estrogen receptor-inducible (tamoxifen-dependent) Cre recombinase (CreER^T2^) under control of the astrocyte-specific glial fibrillary acidic protein (GFAP) promoter (GFAP-CreER^T2^/+) (Fig. 1*B*, right panel). The resulting progeny was GFAP-CreER^T2^/+; mCAT^*LoxP*^/+ and +/+; mCAT^*LoxP*^/+ littermates that, at the age of 8 weeks, were intraperitoneally injected with tamoxifen to activate Cre recombinase activity in astrocytes *in vivo* to generate GFAP-CreER^T2^/+; mCAT/+ (GFAP-mCAT) and +/+; mCAT^*LoxP*^/+ littermates (GFAP-Control) (Fig. 1*B*, right panel). To induce the pro-oxidant neurotoxic challenge, we intraperitoneally injected mice with the neurotoxin 3-nitropropionic acid (3-NP), a well-characterized approach to cause redox stress-mediated striatal degeneration [12,13]. To evaluate the behavioural outcome of the 3-NP insult, we first treated wild type mice with the neurotoxin (or vehicle) and were daily tested for motor coordination in the rotarod test, as depicted in the diagram shown in Fig. 2*D*. 3-NP progressively induced motor impairment (Fig. 2*E*) that correlated with an increase in the MitoSox signal in the brain cells (Fig. 2*F*), suggesting that 3-NP-mediated behavioural impairment is related to excess mROS in the brain.

Since the MitoSox approach may have some limitations [14], and given that the mCAT mouse models herein used specifically target mitochondrial H_2_O_2_, we assessed the effect of 3-NP on mitochondrial H_2_O_2_ *in vivo* using the ratiometric MitoP/B approach [15]. To do so, the MitoB probe [15] was intracerebroventricularly injected in mice immediately after they received injections of 3-NP and vehicle in the right and left hemispheres, respectively [16]. After 6 h, mice were sacrificed and the relative abundances of MitoB and its oxidized product MitoP were assessed by mass spectrometry in each brain hemisphere. As shown in Fig. 2*G*, 3-NP increased the MitoP/B ratio when compared with vehicle within the same mouse, indicating that 3-NP increased brain mitochondrial H_2_O_2_. Interestingly, such an increase in brain mitochondrial H_2_O_2_ was abolished in the 3-NP-treated CaMKIIa-mCAT, but not in the 3-NP-treated GFAP-mCAT mice (Fig. 2*G*). These data strongly suggest that neurons, not astrocytes, are major contributing cells to the excess brain mitochondrial H_2_O_2_ upon 3-NP insult. In good agreement with this notion, the increased apoptotic death of brain cells induced by 3-NP was abolished in CaMKIIa-mCAT, but not in GFAP-mCAT mice (Fig. 2*H*). Accordingly, the loss of motor coordination observed in the wild type mice treated with 3-NP was not detected in CaMKIIa-mCAT and remained in the GFAP-mCAT mice (Fig. 2*I*). These data indicate that selective suppression of mROS enhancement in neurons exhibit neuroprotection against 3-NP-induced degeneration.

## 3 Conclusions

In conclusion, using CaMKIIa-mCAT and GFAP-mCAT mice to modulate redox homeostasis cell-specifically in neurons and astrocytes, respectively, we demonstrate dual roles for mROS in brain physiology and pathology impacting on behaviour (Fig. 2*J*). Thus, according to our results, endogenous basal neuronal mROS abundance does not seem to exert control over mouse behaviour, in contrast with previously reported data [8] showing that astrocytic mROS downmodulation causes cognitive impairment. In this context, here we show that mCAT expression causes a similar degree of mROS reduction (~25%) in both neurons and astrocytes, despite the impact on behaviour is different. In this context, the natural mROS abundance under physiological conditions is considerably higher -about one order of magnitude-in astrocytes than in neurons [6], hence it is conceivable to speculate that any reduction in mROS would have more impact on the redox status of astrocytes affecting behaviour. In contrast, under a pathological condition -such as the 3-NP insult-, here we show that the origin of mROS contributing to brain damage and motor discoordination is neuronal, not astrocytic. These data therefore highlight the importance of targeting specific cell types when addressing the consequences of modulating brain redox homeostasis and organismal behaviour under physiological and pathological conditions. Whether these observations explain the inefficacy of antioxidant therapies that do not selectively target specific cell types, hence failing in clinical trials [17,18], remains to be elucidated. If so, the inducible mCAT^*LoxP*^/+ mouse model herein described may open promising opportunities to validate cell-specific therapies and to understand the biological roles of mROS in health and disease.

## 4 Materials and methods

### 4.1 Animals

All animal procedures were performed according to the European Union Directive 86/609/EEC and Recommendation 2007/526/EC regarding the protection of animals used for experimental and other scientific purposes, enforced in Spanish legislation under the directive RD1201/2005. All protocols were approved by the Bioethics Committee of the University of Salamanca and the Junta de Castilla y Leon (registry number 080). mCAT^*LoxP*/+^ and GFAP-mCAT mice were generated as described [8] and bred under a C57BL/6J background. Primer sequences for genotyping the complete mCAT allele and the ones to recognize Cre-mediated excised allele, devoid of the transcriptional STOP cassette, were previously published [8]. Experiments were performed with male mice at the age of 8–10 months old.

### 4.2 Protein isolation and western blotting

Dissected brain samples or adult brain cell fractions were lysed in RIPA buffer supplemented with phosphatase and protease inhibitors to obtain total protein extracts. Proteins were electrophoretically resolved, transferred into nitrocellulose membranes and immunoblotted with primary antibodies as previously detailed [8]. Primary antibodies were anti-HA tag (1:1000; C29F4, Cell Signaling), anti-β-ACTIN (1:30,000; A5441, Sigma), anti-MAP2 (ab11268, Abcam), anti-PCB (1:1000; ab126707, Abcam) and anti-GAPDH (1:40,000; 4300, Ambion). Secondary antibodies were horseradish peroxidase-conjugated goat anti-rabbit IgG (sc-2030, Santa Cruz Biotechnologies, 1/5000) and goat anti-mouse IgG (1,858,413, Invitrogen, 1/10,000).

### 4.3 Intravenous injections of adeno-associated viral vectors (AAVs)

The AAV-PHP.eB capsids, which efficiently transduce the central nervous system following intravenous injection, were used. These capsids harbour an enhanced green fluorescent protein (EGFP) under the control of neuron-specific human synapsin promoter. AAV-PHP.eB-hSyn-EGFP (Addgene #50465) was administered *via* the retro-orbital venous sinus in anesthetized mice as previously described [8]. Three weeks after infection, brains were extracted and a single-cell suspension was achieved by trypsinization and gentle mechanical trituration, followed by incubation with the dyes for flow cytometric analysis [8].

### 4.4 Immunomagnetic isolation of neuronal cells from adult mouse

Single-cell suspensions from adult brain mouse tissue were obtained following the manufacturer's instructions of the adult mouse brain dissociation kit from Miltenyi Biotec (#130-107-677). Dissociated cells, after removal of debris and red blood cells, were separated with neuron-specific Neuron Isolation Kit (#130-115-389, Miltenyi Biotec) according to the manufacturer's protocol. In summary, brain cells suspension was incubated with biotin-conjugated monoclonal antibodies specific for non-neuronal cells followed by anti-biotin monoclonal antibodies conjugated to MicroBeads. Then, non-neuronal fraction is retained applying a magnetic field allowing the isolation of the unlabelled neurons. We confirmed the identity of the isolated fractions by western blotting against neuronal (microtubule-associated protein 2, MAP2) or astrocytic (pyruvate carboxylase, PCB)-specific markers.

### 4.5 Mitochondrial ROS assessment in brain cells *ex vivo*

Complex samples or immunomagnetically purified single-cell suspensions derived from adult brain mice tissue were incubated with MitoSox to assess overall mitochondrial ROS. We are aware that MitoSox is an unspecific ROS mitochondrial probe [14]. Thus, we refer to ROS to any biologically relevant oxidant that is able to react with this probe, and its choice for part of this study was conditioned by the need to measure the action of mROS in specific brain cell populations in adult mice. Thus, the MitoSox signal in brain cells was evaluated by flow cytometry (FACScalibur flow cytometer, BD Biosciences) in brain cells or GFP^+^ neurons derived from AAV-PHP.eB-hSyn-EGFP injected mice. In all cases, antimycin A (10 μM, Sigma) was used as a positive control.

### 4.6 Mitochondrial membrane potential (Δ**ψ**_m_)

Since the intensity of MitoSox fluorescence may be influenced by the mitochondrial membrane potential, Δψ_m_ was monitored in all samples subjected to MitoSox fluorescence analysis. Δψ_m_ was assessed using the MitoProbe DilC_1_ (1,1′,3,3,3′,3′- hexamethylindodicarbo-cyanine iodide, 50 nM) Assay Kit for flow cytometry (Life Technologies). For this purpose, brain cell suspensions were incubated with the dye at 37 °C for 15 min Δψ_m_ values were expressed in arbitrary units (a.u.). Cells were incubated with CCCP (carbonyl cyanide 4-(trifluoromethoxy) phenylhydrazone, 10 μM) for 15 min and analysed to define the depolarized value (0 Δψ_m_).

### 4.7 Assessment of brain mitochondrial H_2_O_2_

Given the limitations of the MitoSox approach [14], we determined mitochondrial H_2_O_2_ *in vivo* using a ratiometric mass spectrometry probe approach [15]. MitoB ((3-hydroxybenzyl)triphenylphosphonium bromide) contains a triphenylphosphonium cation component that drives its accumulation within mitochondria. MitoB reacts with H_2_O_2_ to form a phenol product, MitoP. Thus, MitoB probe [15] was stereotaxically injected in mice previously anesthetized with sevoflurane (Sevorane; Abbott) using a stereotaxic frame (Model 1900; David Kopf Instruments) with a micromanipulator (Model 1940; David Kopf Instruments) and a digital reading system (Wizard 550; Anilam). MitoB was injected targeting to the lateral ventricle in the coordinates from bregma: anteroposterior: 0.2; medio-lateral: +0.9; dorsoventral: −2), where a small skull hole was performed with a drill (Model 1911; Kopf Instruments). A total of 0.5 nmol (in 2 μl) was administered using a microsyringe attached to a pump (UMP3-1, World Precision Instruments). The infusion took over 4 min, leaving the needle during 5 additional minutes before being slowly removed. Then, mice were sacrificed after 6 h and the brains extracted and dissected. Brain was split through the midline shit and each hemisphere was weighed and snap frozen in liquid N_2_. The samples were processed using the standardized MitoP/MitoB extraction method [15] and internal standards. Thus, each sample was spiked with 10 μl of master mix stock of internal standard (10 μM *d*_*15*_-MitoB 5 μM *d*_*15*_-MitoP) and the LC-MS analysis was performed using a Waters Xevo TQ-S mass spectrometer (Waters, UK). Separation was achieved using an Acquity UPLC BEH 1.7 μM C18 Column (Waters, UK) and the spectra data were processed using MassLynx. *d*_*15*_-MitoB, MitoB, *d*_*15*_-MitoP and MitoP were measured simultaneously by LC-MS/MS. All data were calculated based on MS response relevant to the deuterated internal standards [19]. Results are presented in fold increase, within the same mouse, of MitoP/MitoB ratio values determined for 3-NP-injured hemisphere compared with the contralateral PBS-injected hemisphere.

### 4.8 Behavioural tests

Behavioural tests were performed as previously detailed [8]. Exploration and memory tasks were evaluated using the open-field and novel object recognition test track, registered with the ANY-maze® software and the AMi-maze® interface in the same 40 cm × 40 cm x 35 cm (w, d, h) black infrared transparent Perspex box. Rotarod test (Rotarod apparatus, Model 47,600, Ugo Basile) was used to analyse motor balance and coordination. Mice were previously trained during three consecutive days, i.e. 2 d before the test.

### 4.9 3-Nitropropionic acid (3NP) treatment

This was performed as previously described [13]. In brief, mice received intraperitoneal injections (50 mg/kg of body weight) of 3NP (Sigma-Aldrich, pH 7.4, 0.22 μm-filtered) or vehicle (PBS) every 12 h for a total of seven injections in a volume of 200 μl. Motor coordination of previously trained mice were assessed using the Rotarod test every 24 h since the first 4 h of 3NP treatment. For the acute 3-NP injections, a total of 300 nmol of 3-NP was injected stereotaxically targeting striatum and hippocampus. A volume of 0.5 μl (150 nmol of 3-NP) was injected using a microsyringe attached to a pump (UMP3-1, World Precision Instruments). Each infusion took over 2 min, leaving the needle during 5 additional minutes before being slowly removed. The coordinates from bregma were: anteroposterior: 2; medio-lateral: +/-1.5; dorsoventral: −2 to targeting hippocampus and anteroposterior; +0.5 medio-lateral: +/-2; dorsoventral: −3.3 for striatum. The contralateral hemisphere was injected with the equivalent volume of vehicle (PBS). After 3-NP treatment, mice showed hypoactive, although the hyperactivity rotation was toward the injured side (right). After 6 h, the mice were sacrificed to extract brain and prepare the samples to ratiometric mass spectrometry MitoB approach.

### 4.10 Apoptotic cell death assessment in adult brain cells *ex vivo*

Brain cell suspensions were stained using the combination of APC-conjugated annexin-V (ANXVDY, ImmunoStep) and 7-amino-actinomycin D (7-AAD) (BD Biosciences). Fluorescence intensity was assessed by flow cytometry (FACScalibur flow cytometer, BD Biosciences), measured in arbitrary units and data expressed as fold-change, considering the annexin V^+^/7-AAD^-^ cells apoptotic.

### 4.11 Statistical analysis

For statistical analyses, we used the number of mice indicated in the figure legends. Data are expressed as mean ± standard error of the mean (SEM) values, and the statistical comparisons between two groups of values were performed using the two-tailed Student's *t*-test. In all cases, p < 0.05 values were considered significant. Statistics were performed using Microsoft Excel or the IBM SPSS Statistics software.

## Author contributions

J.P.B. conceived the idea; J.P.B. and C.V.-G. designed research; C.V.-G., N.B., D.J.-B., I.L.-F., G.B. and A.A. performed research; J.P.B., C.V.-G., M.P.M. analysed data; J.P.B. and C.V.-G. wrote the manuscript.

## Declaration of competing interest

The authors declare no competing interest.

## Abbreviations

ROS: Reactive oxygen species
mROS: Mitochondrial reactive oxygen species
mCAT: Mitochondrial-tagged catalase
C8: Cytochrome *c* oxidase subunit VIII–mitochondrial leading sequence
HA: Haemagglutinin
CAG promoter: Cytomegalovirus enhancer fused to the chicken beta-actin promoter
CaMKIIa: Calcium-calmodulin kinase IIa
GFAP: Glial fibrillary acidic protein
ER^T2^: Tamoxifen-inducible estrogen receptor
GFP: Green fluorescence protein
hSyn: human neuron-specific synapsin
3NP: 3-nitropropionic acid
PBS: Phosphate buffered saline
i.p.: Intraperitoneal
7-AAD: 7-amino-actinomycin D
